# Polarity Sensitivity of Human Auditory Nerve Fibers Based on Pulse Shape, Cochlear Implant Stimulation Strategy and Array

**DOI:** 10.3389/fnins.2021.751599

**Published:** 2021-12-08

**Authors:** Amirreza Heshmat, Sogand Sajedi, Anneliese Schrott-Fischer, Frank Rattay

**Affiliations:** ^1^Institute for Analysis and Scientific Computing, Vienna University of Technology, Vienna, Austria; ^2^Laboratory for Inner Ear Biology, Department of Otorhinolaryngology, Medical University of Innsbruck, Innsbruck, Austria

**Keywords:** cochlear implant, computational model, electrical stimulation, finite element model, polarity sensitivity, auditory nerve, pulse shape, cochlear implant stimulation strategy

## Abstract

Neural health is of great interest to determine individual degeneration patterns for improving speech perception in cochlear implant (CI) users. Therefore, in recent years, several studies tried to identify and quantify neural survival in CI users. Among all proposed techniques, polarity sensitivity is a promising way to evaluate the neural status of auditory nerve fibers (ANFs) in CI users. Nevertheless, investigating neural health based on polarity sensitivity is a challenging and complicated task that involves various parameters, and the outcomes of many studies show contradictory results of polarity sensitivity behavior. Our computational study benefits from an accurate three-dimensional finite element model of a human cochlea with realistic human ANFs and determined ANF degeneration pattern of peripheral part with a diminishing of axon diameter and myelination thickness based on degeneration levels. In order to see how different parameters may impact the polarity sensitivity behavior of ANFs, we investigated polarity behavior under the application of symmetric and asymmetric pulse shapes, monopolar and multipolar CI stimulation strategies, and a perimodiolar and lateral CI array system. Our main findings are as follows: (1) action potential (AP) initiation sites occurred mainly in the peripheral site in the lateral system regardless of stimulation strategies, pulse polarities, pulse shapes, cochlear turns, and ANF degeneration levels. However, in the perimodiolar system, AP initiation sites varied between peripheral and central processes, depending on stimulation strategies, pulse shapes, and pulse polarities. (2) In perimodiolar array, clusters formed in threshold values based on cochlear turns and degeneration levels for multipolar strategies only when asymmetric pulses were applied. (3) In the perimodiolar array, a declining trend in polarity (anodic threshold/cathodic threshold) with multipolar strategies was observed between intact or slight degenerated cases and more severe degenerated cases, whereas in the lateral array, cathodic sensitivity was noticed for intact and less degenerated cases and anodic sensitivity for cases with high degrees of degeneration. Our results suggest that a combination of asymmetric pulse shapes, focusing more on multipolar stimulation strategies, as well as considering the distances to the modiolus wall, allows us to distinguish the degeneration patterns of ANFs across the cochlea.

## Introduction

The sophisticated structure of the inner ear provides a sense of hearing obtained by transmitting the auditory signals from cochlear sensory hair cells through the myelinated auditory nerve fibers (ANFs) to the cochlear nuclei in the brain stem. Cochlear implants (CIs) are implanted electronic devices that stimulate surviving ANFs and support impaired auditory pathways to restore a part of a lost hearing sense. Although the CI outcomes depend on individual experiences, dissatisfactions arise when using CI in a noisy environment or listening to music (Gfeller and Lansing, 1991; Leal et al., 2003; Kong et al., 2004; McDermott, 2004; Stickney et al., 2004; Turner et al., 2004; Caldwell and Nittrouer, 2013; Zirn et al., 2016).

Several studies reported considerable variability in outcomes and performances between CI users even by the same audiogram and appropriate listening conditions (Blamey et al., 2012; Holden et al., 2013; Goehring et al., 2017). In addition, various clinical studies investigated the cause of the variability of the outcomes through CI users. They reported factors such as etiology, using hearing aids, age at implantation, ANF degeneration status, surgical trauma, residual hearing measures, electrode displacement in the cochlea, and duration and age at onset of moderate to profound hearing loss have impacts on CI performances and can be the reason for large variations in hearing performance (Gantz et al., 1993; Summerfield and Marshall, 1995; Waltzman et al., 1995; Albu and Babighian, 1997; Shepherd and Javel, 1997; Friedland et al., 2003, 2010; Gomaa et al., 2003; Sly et al., 2007; Finley and Skinner, 2008; Blamey et al., 2012; Lazard et al., 2012; Holden et al., 2013; Frisch et al., 2015; Cosentino et al., 2016). Furthermore, over the decades, it has been shown that sensorineural hearing loss changes ANF geometry by decreasing myelination thickness and ANF diameter or loss of the peripheral part, which negatively affects their excitation properties (Bostock et al., 1983; Colombo and Parkins, 1987; Leake and Hradek, 1988; Spoendlin and Schrott, 1989; Nadol, 1997; Smit et al., 2008; Resnick et al., 2018; Heshmat et al., 2020).

Various studies demonstrate that the neural status is closely related to speech perception in CI users (Khan et al., 2005; Fayad and Linthicum, 2006; Nadol and Eddington, 2006; Kamakura and Nadol, 2016). Detection and identifying ANFs status before implantation are currently unknown and challenging. Any information, such as densities and diameters of ANFs along the cochlear turns, is based on postmortem studies. The deviations from the healthy ear depend on etiology, duration of hearing loss, and so on, and they are quantified in a non-precise way by residual hearing measures.

In recent decades, numerous studies tried to estimate neural health individually in CI users and to find a relationship between implantation outcomes and survival ANFs. Many techniques have been introduced to examine the neural status in CI users, such as pulse rate, pulse polarity, stimulation mode, interphase gap, and phase duration. These techniques revealed substantial outcome variations between subjects and electrodes during psychophysical and electrophysiological measures (Pfingst and Xu, 2004; Bierer, 2007; Bierer and Faulkner, 2010; Bierer et al., 2015a,b; Cosentino et al., 2016; Schvartz-Leyzac and Pfingst, 2016; Zhou, 2016; Mesnildrey et al., 2017, 2019; Carlyon et al., 2018; DeVries and Arenberg, 2018; Guérit et al., 2018). A practical application of postoperative measures is deactivating a single or several electrodes in the array that result in improved outcomes in individual CI recipients (Saleh et al., 2013; Bierer and Litvak, 2016; Vickers et al., 2016; Zhou, 2017); however, increasing the electrode number to more than 8 or 12 helps CI users to have a better perception even in a noisy environment (Croghan et al., 2017; Schvartz-Leyzac et al., 2017). In addition, Zeitler reported that deactivating electrode numbers may cause device failure (Zeitler et al., 2009). Another postoperative approach is computed tomography (CT) imaging analysis of the CI array position inside the cochlea (Saunders et al., 2002; Cohen et al., 2006; Noble et al., 2013, 2014; Long et al., 2014; Van Der Marel et al., 2015; Venail et al., 2015; DeVries et al., 2016), which provides no estimable information about ANF condition and is neither possible nor recommended for all CI users.

Among all the aforementioned techniques, polarity sensitivity is a promising way to estimate neural health based on the difference between negative (cathodic) and positive (anodic) pulse. The concept of polarity sensitivity came from animal studies that reported ANFs are stimulated better by cathodic (CAT) compared with anodic (ANO) pulses, named CAT sensitivity (Hartmann et al., 1984; Miller et al., 1998, 1999). Several computational studies (Rattay, 1999; Rattay et al., 2001a,b; Sajedi et al., 2019) investigated the behavior of ANFs for both polarities and reported inversed sensitivity behavior in humans compared with what is reported in animals. In addition, Rattay demonstrated various action potential (AP) initiation sites of ANFs that differ for CAT and ANO pulse polarities. By applying CAT pulses, peripheral processes are frequently responsible for AP initiation. As the signal needs to pass the large soma with high capacity, much higher threshold currents are needed in degenerated ANFs. In contrast, ANO thresholds are similar in both healthy and degenerated ANFs as long as the initiation sites occur in the central part of the ANF, in which the high capacity of the soma does not need to be loaded (Rattay et al., 2001b; Resnick et al., 2018; Heshmat et al., 2020; Potrusil et al., 2020).

Moreover, similar observations in clinical studies demonstrated that the polarity sensitivity of ANFs is a potential approach to identify neural health in CI users to improve CI performance and enhance speech perception (Macherey et al., 2008; Van Wieringen et al., 2008; Undurraga et al., 2010; Carlyon et al., 2013, 2018; Pfingst et al., 2015a,b; DeVries et al., 2016; Schvartz-Leyzac and Pfingst, 2018; Goehring et al., 2019; Jahn and Arenberg, 2019a; Mesnildrey et al., 2020). On the other hand, several studies on human CI recipients reported different and contradictory outcomes by using polarity sensitivity (Macherey et al., 2006, 2017; Bahmer and Baumann, 2013; Karg et al., 2013; Undurraga et al., 2013; Hughes et al., 2017, 2018; Mesnildrey et al., 2017; Luo J. et al., 2020; Xu et al., 2020). In addition, some studies suggested the variations of polarity sensitivity outcomes occurred not only between CI users but also between CI electrodes, sides of the ear, and cochlear turns (Ranck, 1975; Macherey et al., 2017; Mesnildrey et al., 2017; Carlyon et al., 2018; Goehring et al., 2019; Jahn and Arenberg, 2019a,b). As a consequence of conflicting results from recent investigations, polarity behavior depends on various factors and parameters that might differ in individual CI users. Consequently, the polarity sensitivity of human ANFs still remains unknown and raises some questions about the reliability of this technique and how it can be used to estimate neural health.

Recent clinical studies suggested that pulse configuration and CI stimulation strategies have an impact on the excitation of ANFs (Macherey et al., 2006, 2008, 2017; Bierer, 2007; Bierer and Faulkner, 2010; Undurraga et al., 2010; Bahmer and Baumann, 2012a,b, 2013; Carlyon et al., 2013; Guérit et al., 2018; Hughes et al., 2018; Mesnildrey et al., 2019; Luo X. et al., 2020). However, most present-day CIs stimulate survival ANFs in the monopolar strategy and use symmetric charge balance biphasic pulse, consisting of CAT and ANO polarities. Furthermore, various studies published that the polarity sensitivity could be better investigated with asymmetric pulse shapes such as pseudomonophasic and triphasic (Van Wieringen et al., 2005, 2008; Macherey et al., 2006, 2008, 2017; Bahmer and Baumann, 2013, 2016; Carlyon et al., 2013; Karg et al., 2013; Bahmer et al., 2017; Guérit et al., 2018; Jahn and Arenberg, 2019a). In addition, multipolar stimulation strategies, for example, bipolar and tripolar, can be more efficient for studying polarity sensitivity behavior in human ANFs (Carlyon et al., 2005; Bierer et al., 2011; Bierer and Nye, 2014; Long et al., 2014; DeVries et al., 2016; DeVries and Arenberg, 2018; Jahn and Arenberg, 2019a; Mesnildrey et al., 2019).

Based on the latest investigations, it has been suggested that various parameters may affect the polarity sensitivity of ANFs in CI users. The primary aim of this study is to investigate the impact of some of these parameters on the polarity behavior of ANFs. Therefore, based on our latest clinical study (Heshmat et al., 2020), we considered six groups of ANFs relying on different peripheral diameters and myelination thickness used for computer simulation. Our choice for Rattay’s electric circuit model of ANFs was based on a recent study (Bachmaier et al., 2019), which investigated the three most cited and famous multicompartment models of human ANF and reported that the modified Hodgkin–Huxley model (Rattay et al., 2001b) replicates many features known from experiments in some of their examinations.

Recently, some studies demonstrated that only a detailed cochlear neuron model can replicate a reliable firing pattern, as well as an accurate prediction of AP initiation sites, which plays a crucial role in the AP arrival times at the terminal axonal, known as AP latencies (Bai et al., 2019, 2020). Taking into account the impact of the precise cochlear model, for extracellular stimulation, we adopted an accurate finite element cochlear model and 30 three-dimensional ANF pathways that were reconstructed from tracing real fiber bundles following natural fiber spirality, for example, highly helical pathway in the peripheral sides of the most apical fiber (up to 900° of overall rotation) systematically decreasing in most basal fiber (to 30° overall rotation) (Potrusil et al., 2020).

Furthermore, two CI arrays, one lateral and one perimodiolar, were used in the model from our previous study (Heshmat et al., 2020) to analyze the threshold profiles of the investigated ANFs. By applying symmetric and asymmetric pulses, we studied the impact of the pulse shape on polarity sensitivity. In a second step, we investigated the impact of different CI stimulation strategies. Moreover, because of the difference between lateral and perimodiolar CI arrays, we investigated the effect of the electrode distance to the modiolus wall, as well as the effect of the distance between electrodes. Our final objective was to find an appropriate pattern to explain the variability in polarity behavior and obtain an indicator for polarity sensitivity based on the degeneration status of ANFs.

## Materials and Methods

### Computational Model and Simulations

**FIGURE 1:**
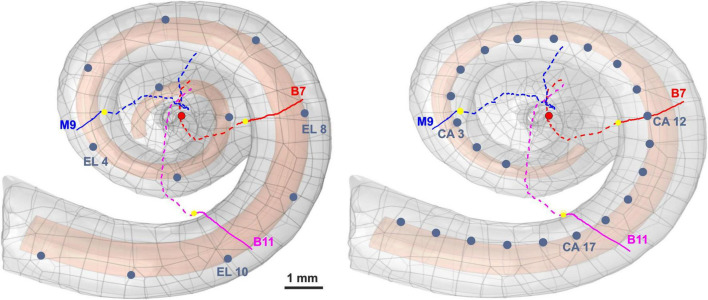
Three-dimensional model of scala tympani including 12 electrodes of a lateral CI (left) and 22 electrodes of a perimodiolar CI (electrodes are close to the center, right), as well as the three investigated pathways of ANFs. The peripheral part, soma position, and central part of the nerve pathways are represented with colored solid lines, yellow spheres, and colored dashed lines, respectively. For the sake of clarity, all electrode positions are shown as dark blue spheres, although in calculations each active electrode is a hemisphere. The axis of modiolus is marked as red circle.

Two electrode arrays, a lateral and a perimodiolar CI, were inserted in scala tympani (Figure 1) of a finite element model based on high-resolution human cochlea micro-CT from our previous work (Potrusil et al., 2020). The geometry included reconstructed pathways of type I ANFs. In the first simulation step, the stimulating voltage profiles along the ANFs were computed for active CI electrode contacts using COMSOL Multiphysics (version 5.5^1^). In the second step, excitations of investigated ANFs were simulated with a multicompartment model (Rattay et al., 2001b). Rattay’s multicompartment model considered the lengths of the nodes of Ranvier, peripheral terminal compartment, and peripheral and central internodes are 2.5, 10, 250, and 500 μm, respectively, except for the last peripheral internode. In addition, the diameter of soma is 20 μm, and the length of the active pre- and post-somatic regions are 100 and 5 μm, respectively. More information can be found in Rattay et al. (2001b) and Potrusil et al. (2020).

The investigated ANFs, named target neurons (TNs), were selected based on the closest distance from their peripheral terminal site to the center of the electrodes. Table 1 represents the angles measured from the round window with respect to the modiolus axis for the six investigated electrodes (three from each CI array system) and TNs in different cochlear turns (basal, middle, and upper–middle). Our previous clinical study (Heshmat et al., 2020) investigated critical morphometry parameters such as diameter and myelination thickness of the peripheral parts of ANFs for several human cochleae at various ages with different hearing loss levels. According to observations of different distributions in diameter and myelination thickness, the study suggested a degeneration pattern for peripheral parts of human ANFs based on hearing loss levels. Therefore, the peripheral process morphometry was divided into six groups indicating different neural health statuses (Table 2) according to the different varieties of the peripheral parts of human ANFs.

**TABLE 1:** Angles of the investigated electrodes and the corresponding target neurons (TN) measured from the round window with respect to modiolus axis; compare Figure 1.

Lateral electrode array	Electrode angle (degrees)	Perimodiolar electrode array	Electrode angle (degrees)	TN	TN angle (degrees)
EL4	343	CA3	332	M9	327
EL8	143	CA12	153	B7	137
EL10	69	CA17	73	B11	79

**TABLE 2:** Six ANFs status based on degeneration levels of peripheral diameter.

ANF state	Degeneration levels	Peripheral diameter (μm)
AS1	Intact	2
AS2	Slight	1.5
AS3	Moderate	1
AS4	Severe	0.75
AS5	Profound	0.5
AS6	Progressive	Without peripheral

**FIGURE 2:**
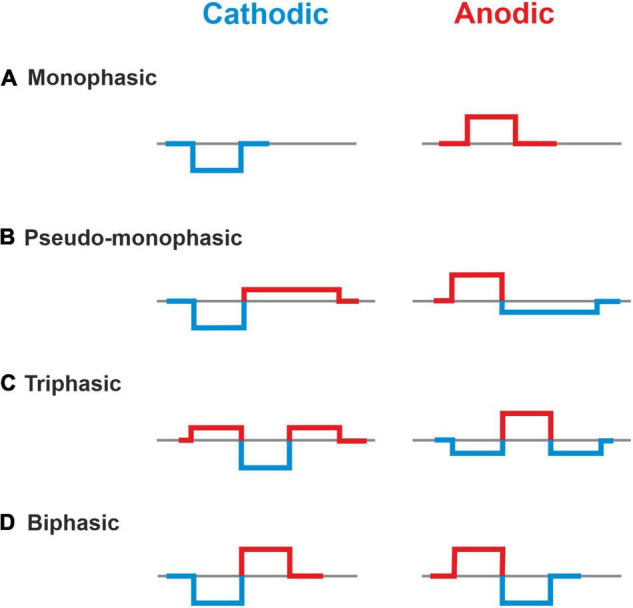
Investigated asymmetric and symmetric pulse shapes. **(A)** Monophasic, **(B)** Pseudo-monophasic, **(C)** Triphasic, and **(D)** Biphasic pulse. Blue and red colors represent the cathodic and anodic phases, respectively.

Monophasic, pseudomonophasic, triphasic, and biphasic pulses were applied for both polarities, CAT and ANO (Figure 2). The phase duration was 100 μs, except for the second phase of pseudomonophasic pulse, in which the duration was increased by a factor of four, and its amplitude decreased by the same factor to keep the charge balanced (Macherey et al., 2011). All multicompartment model evaluations were performed in MATLAB (version R2020a^2^).

**FIGURE 3:**
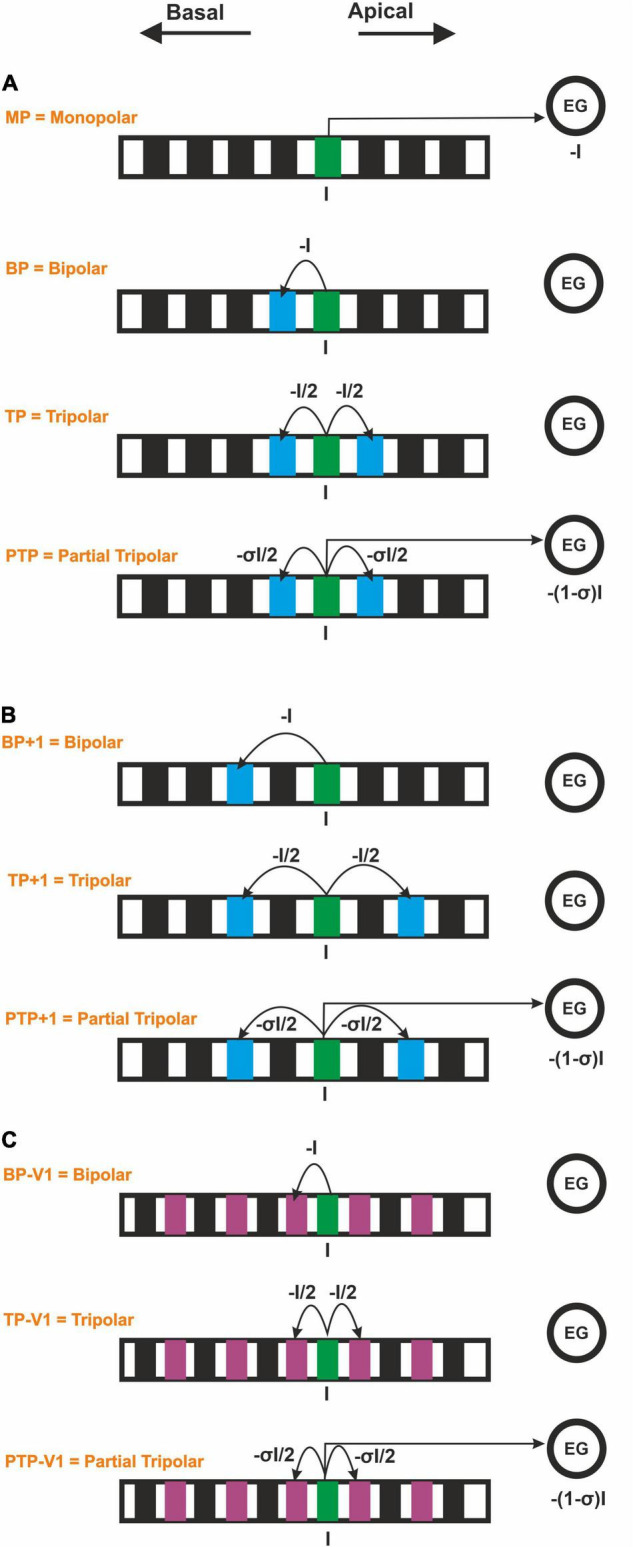
Forced current flow (I) from the stimulating CI channel for monopolar, bipolar, tripolar, and partial tripolar configuration. **(A)** Monopolar strategy and multipolar strategies by considering the basal adjacent channel. **(B)** Multipolar strategies by considering the second basal adjacent channel in perimodiolar CI. **(C)** Multipolar strategies by considering a shorter interchannel distance in the lateral CI.

Moreover, four CI stimulation strategies, which vary depending on the return electrode (ground electrode) position, were used in this study: (i) a monopolar strategy (MP), with the return electrode located outside the cochlea, usually behind the ear; (ii) a bipolar strategy (BP), with the return electrode placed next to the stimulating electrode; (iii) a tripolar strategy (TP) that the return electrodes are two neighboring electrodes, each electrode taking half of the current delivered to the stimulating electrode; and (iv) a partial tripolar strategy (PTP) with a combined configuration of MP and TP, in which part of the stimulating current (defined by the coefficient σ, 0 < σ < 1) returns to the two neighboring electrodes and the rest to the common ground (Figure 3). The investigated PTP configuration was considered with 75% of the current returning to the neighboring electrodes and 25% to the ground, according to Jolly et al. (1996); Litvak et al. (2007), and Mesnildrey et al. (2020). These four CI stimulation strategies (MP, BP, TP, and PTP) were evaluated using COMSOL (Figure 3) for both CI systems.

Two geometrical parameters are important in order to compare the lateral and the perimodiolar CI: (i) the center-to-center distance of adjacent channels and (ii) the distance to the modiolus axis. To perceive the impact of each of these parameters, first, we used the CI systems with features as reported by the manufacturer. Next, we applied a similar distance between channels in both CI arrays and compared the results with the original arrays. For this aim, in perimodiolar CI, we considered the second basal adjacent channels (Figure 3B), whereas, in lateral CI, an extra electrode was set between two investigated electrodes each time, which was used as the new ground electrode (Figure 3C).

## Results

### Impact of Type of Array, Pulse Shape, Stimulus Strategy, and Degeneration Level on Threshold Current

**FIGURE 4:**
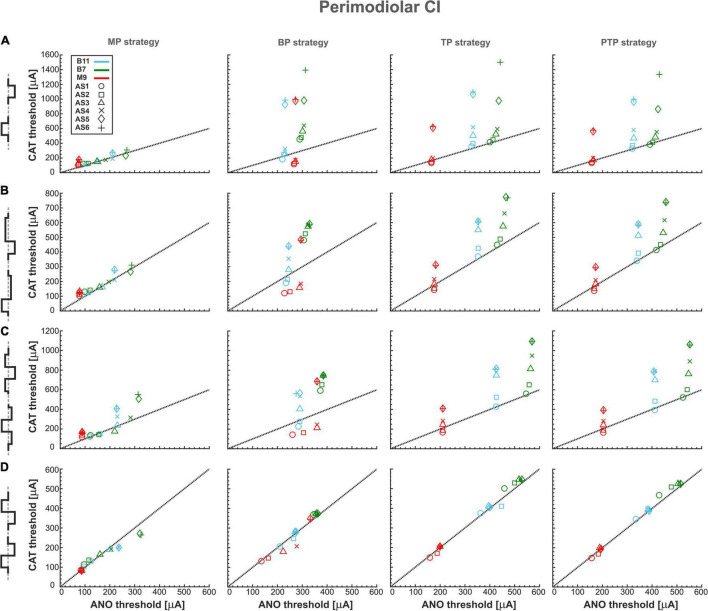
Threshold profiles of three TNs for perimodiolar CI. Thresholds are shown for four stimulation configurations: MP, BP, TP, and PTP with **(A)** monophasic, **(B)** pseudomonophasic, **(C)** triphasic, and **(D)** biphasic pulse. Blue, green, and red colors represent B11, B7, and M9, respectively, in all panels. ANF status based on degeneration levels is indicated with circle, rectangle, triangle, cross, diamond, and plus shapes for AS1, AS2, AS3, AS4, AS5, and AS6, respectively. Black dotted lines represent the ANO/CAT = 1. Legend from top-left panel A applies to all panels.

Figure 4 summarizes excitation thresholds of three investigated TNs in the basal, middle, and upper–middle turn for the perimodiolar CI with pulse shapes: monophasic, pseudomonophasic, triphasic, and biphasic in the MP and three multipolar strategies: BP, TP, and PTP. In all panels, six ANF statuses based on degeneration levels (Table 2) were considered with a circle (intact), rectangle, triangle, cross, diamond, and plus, for AS1, AS2, AS3, AS4, AS5, and AS6, respectively.

Figure 4A shows the excitation thresholds of the monophasic pulse for three TNs, B11 (blue), B7 (green), and M9 (red), in four stimulation strategies. The MP strategy needs the lowest currents in both polarities. The deviation between ANO and CAT thresholds is small for each degeneration case. This is obvious as most cases accumulated over the black dotted line where the polarity ratio (ANO/CAT) is 1. In addition, the threshold increases in both polarities by increasing the degeneration levels of TNs except in M9, which has almost the same ANO threshold for all degenerated levels because, in all status cases, AP initiation site is the central process.

Moreover, in contrast to MP strategy, the ANO threshold ranges increase negligibly in BP, TP, and PTP; however, the CAT threshold ranges increase significantly. The investigated TNs have almost equal ANO thresholds for different ANF degenerated cases, which indicates the AP initiates from nearly the same site of the central process of TNs. In contrast, the CAT threshold variations are substantial and increased significantly in each level of degeneration, especially in AS5 and AS6 cases. Finally, the behaviors of the fibers in TP and PTP strategies are similar to each other. To summarize, using monophasic pulse in four stimulation strategies shows that all threshold cases are densely accumulated in MP, whereas, in multipolar strategies, each TN individually forms a cluster that can be distinguished by degeneration levels.

**FIGURE 5:**
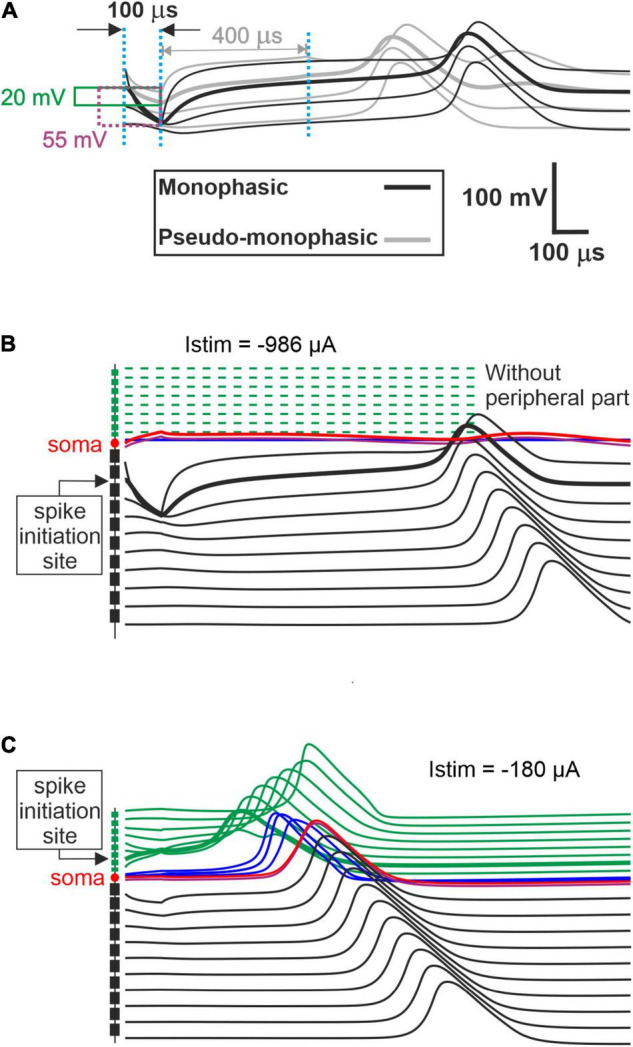
Comparison of the transmembrane voltage just above the threshold. **(A)** Comparison between excitation of the initiation sites for both pulse shapes. During the first cathodic phase, the strongest deviations from the resting voltage appear in a central process region close to the soma. Hyperpolarization of the monophasic pulse (55 mV) is almost three times larger than for the pseudomonophasic pulse (20 mV). In monophasic stimulation, the central process compartment with the strongest hyperpolarization elicits the spike by break excitation (thick black line), and this spike is conducted in both directions (thin black lines). Break excitation is an overshoot of membrane voltage in the direction of the resting potential after a strong hyperpolarization. This overshooting trend is strongly supported by the weak second phase of the pseudomonophasic pulse (gray lines). Consequently, the pseudomonophasic stimulation needs less hyperpolarization during the first phase. **(B)** Degeneration status AS6, without peripheral process, when monophasic pulse is applied. **(C)** Excitation of fiber with healthy status for monophasic pulse.

Figure 4B represents the excitation thresholds for the pseudomonophasic pulse. The ANF behavior is almost similar to monophasic pulse (Figure 4A) for all strategies. Furthermore, the ANO threshold variations in the BP, TP, and PTP are minimal between degeneration cases of individual TNs compared with MP strategy, and again, the clustering behavior is formed in multipolar strategies compared with MP. Interestingly, the CAT threshold ranges are decreased by half compared with monophasic pulse, as in Figure 4A. This threshold difference can be understood from the voltage membrane changes induced by the two pulse shapes, as shown in Figure 5A. The pseudomonophasic pulse has a lower negative slope at the onset of the CAT-leading phase compared with the monophasic pulse represented with the pink rectangle (Figure 5A). Therefore, the pulse makes the transmembrane voltage less negative (20 vs. 55 mV hyperpolarized). The second charge-balanced phase of the pseudomonophasic pulse has a positive effect, causing the transmembrane voltage to depolarize more and faster to the excitation threshold level. For a better understanding, the voltage membrane changes by applying a monophasic pulse are displayed over time for a progressively degenerated fiber (Figure 5B) and a healthy fiber (Figure 5C).

Figure 4C demonstrates the excitation thresholds with the triphasic pulse. The ANF behavior is almost comparable to previous pulse shapes in all stimulation strategies, and again, the clustering behavior is formed in multipolar strategies compared with MP. Moreover, in multipolar strategies, the CAT threshold ranges of the triphasic pulse are approximately 50% higher than pseudomonophasic and 50% lower than the monophasic pulse.

Figure 4D displays the excitation thresholds with biphasic pulse. The behavior in the MP strategy is similar to the previous pulse shapes but with less variability and polarity ratio almost close to 1. However, the cluster form based on degeneration levels among individual TNs is not visible anymore. The TN thresholds mostly accumulate over the black dotted line, resulting from the same AP initiation site of TNs in different degeneration levels in both polarities. As a result of losing the CAT threshold variations in multipolar strategies by applying the biphasic pulse, it is challenging to distinguish TNs response based on different degeneration levels, which differs from the previous pulse shapes (Figures 4A–C).

**FIGURE 6:**
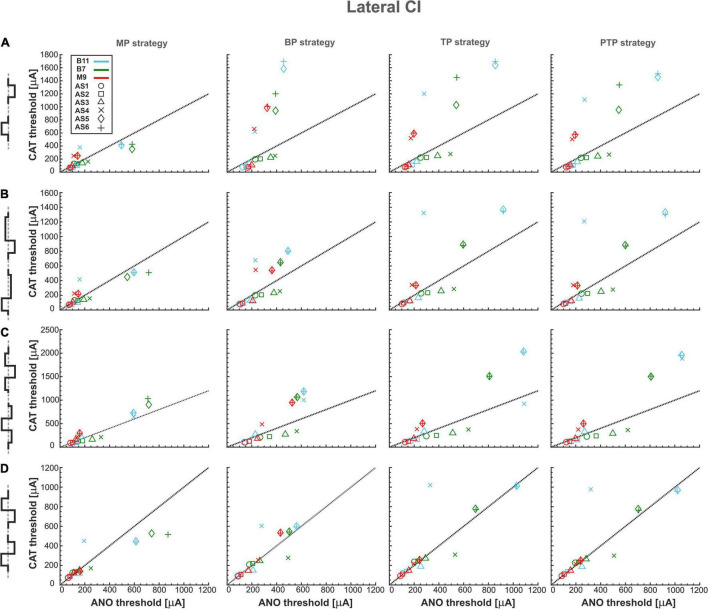
Threshold profiles of three TNs for lateral CI. Layout as in Figure 4.

Figure 6 shows excitation thresholds as Figure 4 but for a lateral CI. Subsequently, Figure 6A displays the excitation thresholds of monophasic pulse for the three investigated TNs in the four stimulation strategies. The MP strategy needs significantly lower currents, especially in CAT thresholds, compared with the other strategies. In addition, the ANO and CAT thresholds do not change substantially by increasing degeneration levels. However, both ANO and CAT thresholds increase significantly in multipolar strategies compared with the MP strategy. The ANO threshold variations between the TNs are small in the BP strategy compared with TP and PTP strategies. However, the cluster behavior is not formed in multipolar strategies in lateral CI, as they formed in the perimodiolar CI (Figure 4A).

Figure 6B represents the excitation thresholds with the pseudomonophasic pulse. The trend is similar to monophasic pulse (Figure 6A) for all strategies, although CAT thresholds in the BP are significantly lower and more concentrated compared with monophasic pulse in severe degenerated cases (Figure 6A, BP strategy). Surprisingly, the similar CAT threshold ranges between monophasic and pseudomonophasic pulses (Figures 6A vs. 6B) are in contrast to the CAT threshold behavior in the perimodiolar CI case (Figures 4A vs. 4B).

Figure 6C displays the excitation thresholds with the triphasic pulse with a similar structure as Figures 6A,B. The ANF behavior is comparable to previous asymmetric pulses, especially with pseudomonophasic. The CAT threshold ranges of triphasic pulses are approximately 25% larger than monophasic and pseudomonophasic pulses.

Figure 6D demonstrates the excitation threshold with the biphasic pulse. The ANO and CAT threshold ranges are almost doubled compared with the perimodiolar case (Figure 4D). The threshold characteristics in both polarities are comparable in all four stimulation strategies for all TNs, except in some severe degenerated cases.

### Impact of Type of Array, Pulse Shape, Stimulus Strategy, and Degeneration Level on Polarity Ratios and Polarity Sensitivity

**FIGURE 7:**
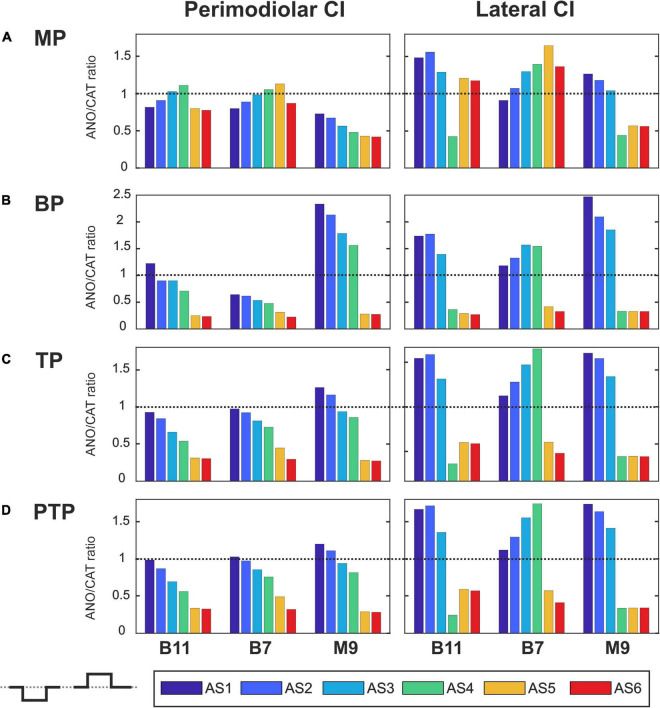
Polarity ratios for different degeneration levels stimulated with the monophasic pulse. Threshold ratios are shown for the TNs B11, B7, and M9 with color-coded degeneration levels **(A)** monopolar (MP), **(B)** bipolar (BP), **(C)** tripolar (TP), **(D)** partial tripolar (PTP) stimulation strategies. The horizontal dotted lines indicate the polarity ratio ANO/CAT = 1.

**FIGURE 8:**
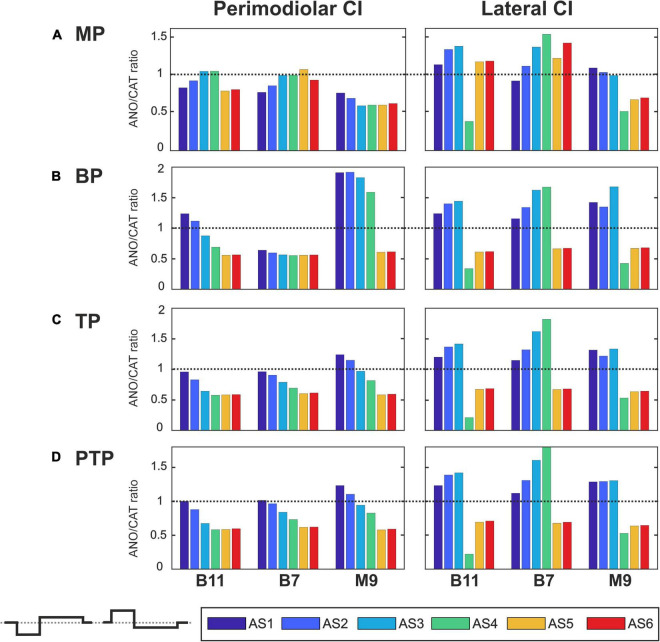
Polarity ratios for different degeneration levels stimulated with the pseudomonophasic pulse. Layout as in Figure 7.

**FIGURE 9:**
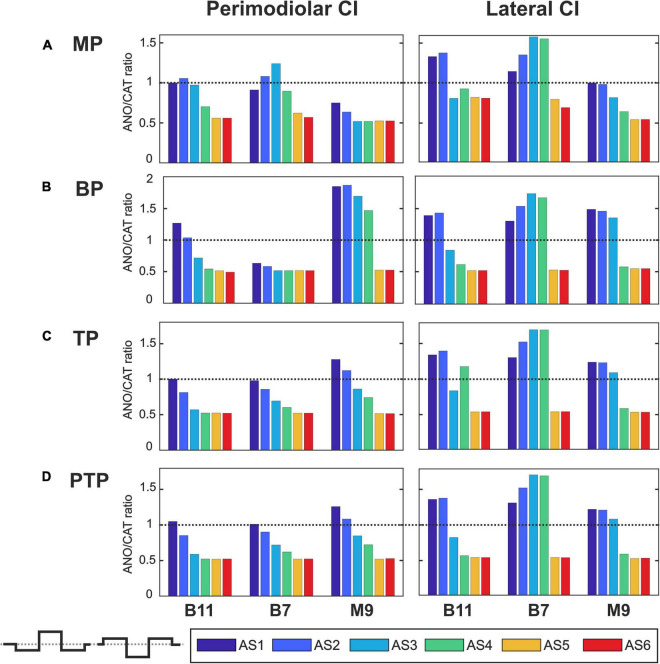
Polarity ratios for different degeneration levels stimulated with the triphasic pulse. Layout as in Figure 7.

**FIGURE 10:**
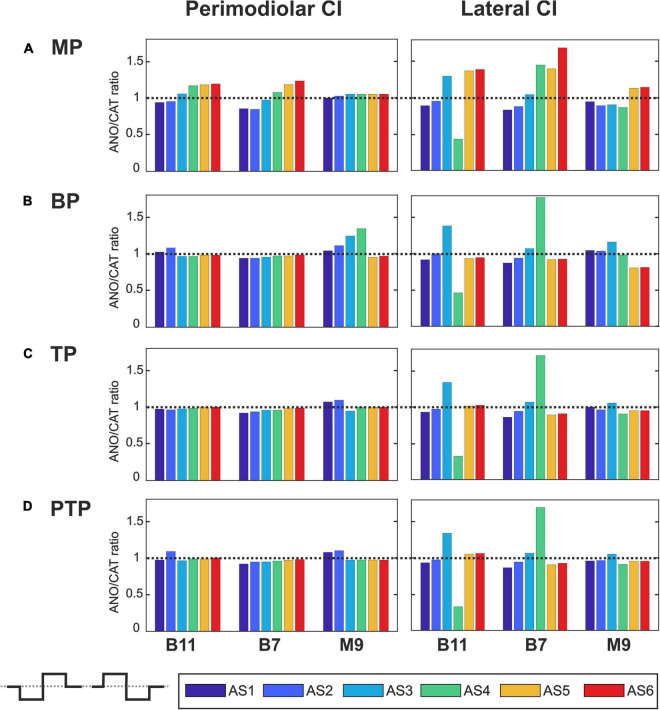
Polarity ratios for different degeneration levels stimulated with the biphasic pulse. Layout as in Figure 7.

Figures 7–10 summarize the polarity sensitivity defined by the ANO/CAT threshold ratios of three investigated TNs for the perimodiolar CI (left panels) and lateral CI (right panels) with pulse shapes: monophasic (Figure 7), pseudomonophasic (Figure 8), triphasic (Figure 9), and biphasic (Figure 10), in the MP and the three multipolar strategies for the six degeneration levels (Table 2). The trend ANO/CAT < 1, indicating the lower ANF threshold for anodic stimulation, is related to the degeneration level and the modiolus distance of the stimulating electrode. Polarity sensitivity is much more pronounced for monophasic (Figure 7), pseudomonophasic (Figure 8), and triphasic (Figure 9) versus biphasic (Figure 10) stimulation.

Figure 7 illustrates the polarity threshold ratios with monophasic pulse for two CI systems. In the MP strategy (Figure 7A), ANO sensitivity (ANO/CAT < 1) in the perimodiolar CI occurred more frequently except in four cases that can be related to nearer-distance electrode to modiolus wall. A declining trend in ratios appears from intact to more degenerated cases in both CI array systems only for the upper middle TN, M9.

Figures 7B–D display the polarity ratios in multipolar strategies for both CI systems. Interestingly, a declining trend in ratios always happens for all three TNs in the case of the perimodiolar array. More degenerated cases (AS4–AS6) become ANO sensitive in the lateral system, whereas CAT sensitivity happens in intact and less degenerated cases for all three multipolar strategies. However, in the perimodiolar array, regardless of degeneration level, most cases show ANO sensitivity.

Figure 8 shows the polarity threshold ratios for pseudomonophasic pulse. The behavior is very similar to monophasic pulse (Figures 7A–D). Interestingly, again the decreasing trend can be observed in the perimodiolar array for all TNs in the three investigated multipolar strategies. In contrast, in the lateral array system, ANO versus CAT sensitivity occurs mainly in AS4–AS6 degenerated cases versus intact, slight, and moderate degenerated cases (AS1–AS3).

Figure 9 demonstrates the polarity threshold ratios for triphasic pulse. The behavior is similar to monophasic and pseudomonophasic pulses (Figures 7, 8). Again, the decreasing trend can be noticed in the perimodiolar array for all TNs in the three investigated multipolar strategies. In contrast, in the lateral array system, ANO versus CAT sensitivity occurs mainly in AS4–AS6 vs. AS1–AS3.

Figure 10 shows the polarity threshold ratios with biphasic pulse for both CI systems. Contrary to the previous pulses, for the biphasic pulse in the lateral array, the ANO sensitivity of the high degeneration versus CAT sensitivity for intact and less degenerated cases is not observed anymore. In most cases of the perimodiolar array, the polarity ratio is close to 1; hence, the declining behavior in perimodiolar CI from intact to progressive degeneration does not occur in the biphasic pulse.

### Impact of Type of Array, Pulse Shape, Pulse Polarity, Stimulus Strategy, and Degeneration Level on Action Potential Initiation Sites

**FIGURE 11:**
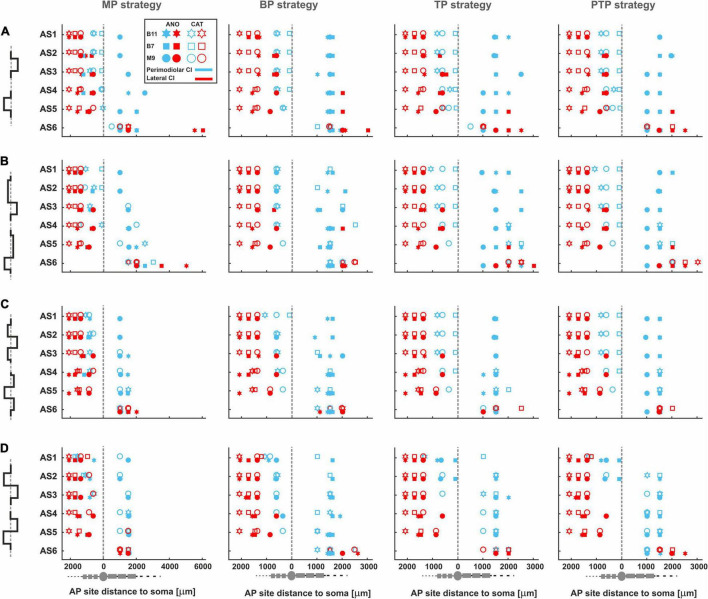
AP initiation sites at ANO and CAT thresholds for four pulse shapes in four stimulation strategies. The TNs, B11, B7, and M9, are demonstrated with star, rectangle, and circle, respectively. In addition, the lateral and perimodiolar CI systems are indicated with red and blue colors. Pulse polarity is identified by filled (ANO) and unfilled shapes (CAT). Gray dashed lines show the soma positions. AP initiation sites for **(A)** monophasic, **(B)** pseudomonophasic, **(C)** triphasic, and **(D)** biphasic pulse. Legend from top-left panel A applies to all panels.

Figure 11 displays AP initiation sites at ANO and CAT threshold levels for the three investigated TNs in the basal, middle, and upper–middle turn for the perimodiolar CI (blue) and the lateral CI (red) with pulse shapes: monophasic (A), pseudomonophasic (B), triphasic (C), and biphasic (D) in the MP and the three multipolar strategies. In all panels, *y* axis represents the six ANF statuses based on the degeneration levels, as in Table 2.

Figure 11A shows AP initiation sites when a monophasic pulse is applied. In the lateral CI, AP mostly initiates at the peripheral sites regardless of the pulse polarity, stimulation strategy, and degeneration levels, except for progressive cases, which are considered without peripheral process. However, in the primordial CI, the AP initiates in the periphery when CAT pulse is applied except for the progressive cases, and in the case of ANO pulses, the initiation sites mainly occur in the central processes.

Figures 11B–C display the AP initiation sites when pseudomonophasic and triphasic pulse is applied. The same behavior in AP initiation sites can be observed in the lateral array as in Figure 11A, although by using the perimodiolar array, initiation sites differ for ANO versus CAT thresholds mostly when TP and PTP strategies are used.

By applying a symmetric biphasic pulse (Figure 11D), the lateral CI keeps the AP initiation sites in the peripheral sites, whereas in the perimodiolar array, the AP initiation sites are mostly the same in both CAT and ANO thresholds regardless of TN degeneration levels.

### Impact of Distance Between Channels and Distance to Modiolus Wall on Polarity Sensitivity

Differences in threshold characteristics between perimodiolar and lateral CIs result from the electrode to modiolus distance and the distance between channels. To investigate the impact of distance between channels, one channel gap is considered in perimodiolar CI by increasing the distance between channels from 0.7 to 1.4 mm (Figure 3B). In addition, for the lateral CI, we decreased the distance between channels from 2.4 to approximately 1.3 mm (Figure 3C).

**FIGURE 12:**
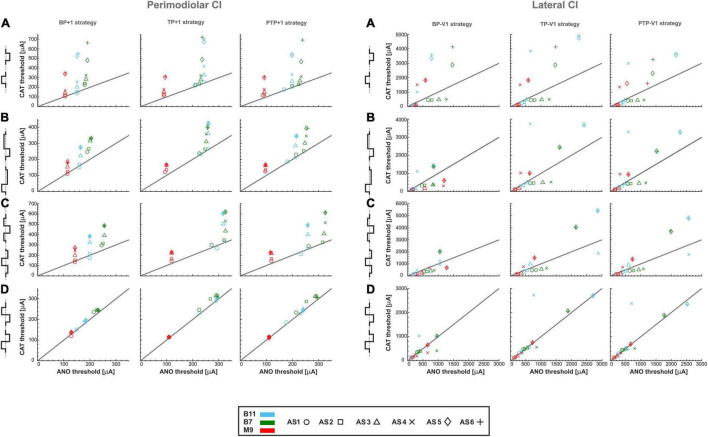
Threshold of three TNs for perimodiolar CI (left panel) and lateral CI (right panel) for four different pulse types and three multipolar strategies by changing the distance between channels of both CI systems. **(A)** monophasic, **(B)** pseudomonophasic, **(C)** triphasic, and **(D)** biphasic pulse. Layout as in Figure 4.

The effect of doubled channel separation is evident for multipolar configurations in perimodiolar array; compare Figures 4, 12, left panels. For monophasic pulses, the ANO and CAT threshold ranges are reduced by a factor of 2 when the distance of returning electrode is increased (Figures 12A, left panel vs. 4A). The clustering behavior is also noticeable in all three multipolar strategies. The investigated TNs have individually similar ANO thresholds in all degenerated cases; in contrast, the CAT thresholds are increased in each level of degeneration, similar to that in Figure 4A but with lower thresholds (Figure 12A, left panel).

The left panel of Figure 12B shows similar characteristics of pseudomonophasic pulses as in monophasic pulse but with lower CAT thresholds dropped by half (Figures 12A vs. B, left panels), as Figures 4A,B for multipolar strategies. The same clustering behavior can be observed as in Figure 4B.

Triphasic pulse (Figure 12C, left panel) displays similar behavior with previous pulses with the almost same CAT threshold ranges as in monophasic pulse (Figure 12A, left panel) and higher CAT thresholds than pseudomonophasic pulse (Figure 12B, left panel). In addition, the ANO and CAT threshold ranges are reduced by a factor of 2 when the distance of returning electrode is increased compared with Figure 4C.

For biphasic pulse again, the ANO and CAT threshold ranges in the multipolar strategies are decreased by a factor of about two (Figures 12D, left panel vs. 4D). However, the cluster form based on degeneration levels among individual TNs is not visible anymore, and the threshold ratios are mostly close to one, as in Figure 4D. Overall, both polarity threshold ranges are decreased by increasing the returning electrode distance in all multipolar strategies, which are related directly to current shunting between electrodes.

The effect of halving channel distance is studied and shown in Figure 12, right panels for multipolar strategies in the lateral CI. As displayed in the right panels of Figures 12A–D, the threshold characteristics are very similar to multipolar strategies compared with Figures 6A–D, except for the threshold ranges, which are significantly increased. The ANO/CAT ratios are again close to 1 (Figures 6D, 12D, right panel) for the biphasic pulse, and no cluster is formed regardless of pulse shape and stimulus strategies as before.

**FIGURE 13:**
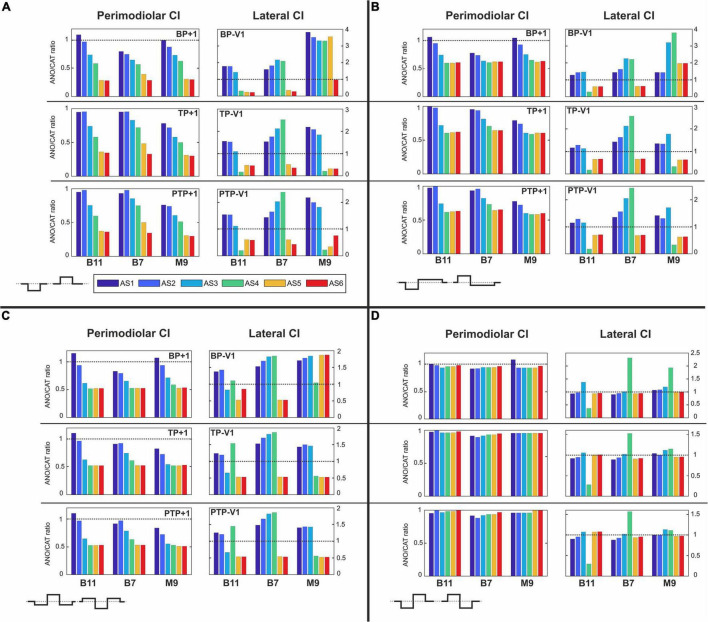
Polarity ratios for different degeneration levels stimulated with **(A)** monophasic, **(B)** pseudomonophasic, **(C)** triphasic, and **(D)** biphasic pulse by changing the distance between channels of both CI systems. The horizontal dotted lines show the polarity ratio is equal to one (ANO/CAT = 1). Legend in **(A)** applies to all panels.

Figure 13A represents the polarity ratios for monophasic stimulation. The polarity ratio in perimodiolar CI significantly decreased by doubling the distance between channels compared with Figures 7B–D, left panels. Interestingly, the declining trend is also again visible between intact and different degenerated cases, similar to Figures 7B–D (left panels). In contrast, the polarity ratio substantially increased by halving the distance between channels in the lateral CI (Figure 13A, right panels). However, the polarity behavior of all TNs in intact and different degenerated levels is similar to Figures 7B–D (right panels).

Figure 13B illustrates the polarity ratios with pseudomonophasic stimulation. The polarity ratio is significantly decreased by doubling the distance between channels in perimodiolar CI (Figure 13B, left panels) compared with Figures 8B–D (left panels). The declining trend is again visible between intact and most degenerated cases, similar to Figures 8B–D (left panels). On the other hand, lateral CI shows similar polarity behavior as monophasic pulse (Figure 13A, right panels) and Figures 8B–D, right panels.

Figure 13C demonstrates the polarity ratios for triphasic stimulation. In perimodiolar CI (Figure 13C, left panels), the polarity ratio is significantly decreased by doubling the distance between channels compared with Figures 9B–D (left panels). Again, the declining trend is noticeable between intact and most degenerated cases, similar to Figures 9B–D, left panels. Lateral CI represents similar polarity behavior as previous pulses (Figures 13A,B, right panels; Figures 9B–D, right panels).

Figure 13D shows the polarity ratios for biphasic stimulation. In contrast to the previous pulse shapes, by applying the biphasic pulse in the lateral CI array (Figure 13D, right panels), the polarity effect is lost, similar as in Figures 10B–D, right panels. The declining trend in perimodiolar CI (Figure 13D, left panels) from intact to progressive degeneration does not occur by applying this pulse shape, same as in Figures 10B–D, left panels.

## Discussion

### Effect of Pulse Shape

Biphasic symmetric charge balance pulse is widely used as a standard pulse shape to stimulate the surviving ANFs in CIs. According to numerous clinical studies, pulse shape is a critical factor for studying neural status (Undurraga et al., 2010; Bahmer and Baumann, 2012a,b, 2013; Hughes et al., 2018; Luo J. et al., 2020). Consistent with several clinical studies, which reported that asymmetric pulse shapes are more effective than symmetric biphasic pulse for evaluating polarity sensitivity in CI users (Macherey et al., 2006, 2008, 2017; Carlyon et al., 2013; Guérit et al., 2018; Jahn and Arenberg, 2019a), our results showed that when a symmetric biphasic pulse is applied, recognition of peripheral changes based on polarity sensitivity was not feasible. We found threshold variations, and consequently, polarity sensitivity between degeneration levels could not be detected with symmetric biphasic pulses. As a symmetric biphasic pulse consists of phases with equal amplitude and duration but opposite polarities, the contribution of the extracellular gradient is almost the same for both phases; consequently, it may not be possible to estimate which polarity affects the ANFs mainly.

In agreement with the presented results, several studies reported that symmetric biphasic pulses did not provide much evidence for polarity sensitivity (Undurraga et al., 2013; Guérit et al., 2018; Hughes et al., 2018; Xu et al., 2020). In addition, Spritzer groups found opposite results of polarity, which was consistent with (Macherey et al., 2006), and also, they could not find polarity sensitivity for peak electrode locations (Spitzer and Hughes, 2017; Spitzer et al., 2019). They suggested that using symmetric biphasic pulse is not an excellent choice to study polarity sensitivity (Spitzer et al., 2019). In line with findings by Undurraga et al. (2013) who reported the same ANO and CAT threshold detection using biphasic pulse, we found ANO/CAT ratios mostly close to one in perimodiolar array when a biphasic pulse was applied to the active electrodes.

In contrast to symmetric pulses, several studies demonstrated promising results in polarity investigations by using asymmetric pulse shapes, for example, pseudomonophasic and triphasic (Macherey et al., 2006, 2008, 2017; Carlyon et al., 2013, 2018; Undurraga et al., 2013; Bahmer et al., 2017; Mesnildrey et al., 2017; Jahn and Arenberg, 2019a). However, some of these studies could not provide direct evidence to prove the role of the polarity effect in estimating neural health, which might result from various factors such as stimulation strategy and CI systems.

Despite using triphasic pulse, Carlyon et al. (2018) and Jahn and Arenberg (2019a) reported that up to 70% of investigated electrodes showed anodic sensitivity and could not prove polarity behavior as an indicator for estimating neural survival. Similar to their results, we found more anodic sensitivity using asymmetric pulses, such as pseudomonophasic and triphasic pulses, in the perimodiolar array. However, this anodic sensitivity occurred not only in degenerated cases but also in intact case; because the perimodiolar array is mostly close to the modiolus axis, anodic pulses may exhibit more efficiency as they active central parts of ANFs (Figure 11). On the other hand, we found a reduction trend in polarity ratios from approximately 1 in intact to approximately 0.5 in the most degenerated case. As anodic sensitivity is observed more frequently regardless of neural health status, we suggest that considering the ANO/CAT ratios and comparing them across the array might shed light on estimating neural health in future investigations.

### Effect of Cochlear Implant Stimulation Strategy

Several clinical investigations demonstrated that MP strategy induces uniform thresholds across the CI and develops outcomes with poor tonotopic representation due to a broad electric field (Bierer and Middlebrooks, 2002; Bierer et al., 2015a), yet MP stimulation strategy is widely used as a standard strategy in commercial CI systems. In contrast, multipolar strategies, such as BP, TP, and PTP, produce sharper electric fields compared with the MP strategy (Kral et al., 1998; Bierer and Middlebrooks, 2002; Snyder et al., 2004; Nelson et al., 2008; Bierer and Faulkner, 2010; Landsberger et al., 2012; Srinivasan et al., 2013; Long et al., 2014). Evidence from numerous psychophysical and physiological studies suggests that multipolar strategies increase selectivity, reduce channel interaction, and cause larger variation of detection thresholds between electrodes across CI array, and it is beneficial for diagnostic purposes, particularly for investigating the electrode neuron interface (Pfingst and Xu, 2004; Bierer, 2007; Bierer and Faulkner, 2010; Landsberger et al., 2012; Srinivasan et al., 2013; Bierer and Nye, 2014; Long et al., 2014; Schvartz-Leyzac et al., 2020). Undurraga et al. (2012) and Chatterjee and Kulkarni (2014) have reported that multipolar strategies cause changes in AP initiation sites leading to threshold variation. In agreement with the findings mentioned previously, we found the obtained threshold values were more concentrated and in the same range in MP stimulation, whereas by applying multipolar strategies, threshold values created clusters based on different degeneration levels as well as cochlear turn.

While degeneration of ANFs occurs mostly in the peripheral part (Fayad and Linthicum, 2006), peripheral processes may remain partly intact. However, depending on hearing loss levels, reduction in diameter and myelination thickness happens (Spoendlin and Schrott, 1989; Heshmat et al., 2020). These findings suggest that variation in peripheral processes is imminent; thus, peripheral excitation results in behavioral differences based on degeneration status across the array.

Various clinical studies had suggested that polarity effects were observed more significantly when multipolar strategies were applied (Macherey and Carlyon, 2010; Macherey et al., 2011; Undurraga et al., 2012; Jahn and Arenberg, 2019a; Mesnildrey et al., 2020). Miller and Chatterjee have reported that multipolar strategies excite more peripheral processes of ANFs and demonstrate better local ANF activation and degeneration patterns (Miller et al., 2003; Chatterjee and Kulkarni, 2014). In alignment with those studies, our results showed that with changing the level of degenerations in the peripheral process, at least for one polarity, threshold values were changed based on peripheral degeneration levels when multipolar strategies were used. Consequently, in the perimodiolar CI, the polarity threshold ratios significantly differed and followed a declining trend for intact to progressive degenerated cases, whereas by using MP stimulation, the declining trend was lost. In addition, in the lateral CI, the anodic versus cathodic sensitivity occurred mainly in severe to progressive degenerated cases versus intact to moderate cases by applying only multipolar strategies. However, according to our results, applying symmetric pulse shape regardless of stimulation strategy led to similar threshold ranges in polarity, i.e., polarity threshold ratios close to 1 (Figure 10), which agrees with clinical studies (Macherey et al., 2008, 2006; Undurraga et al., 2013). Furthermore, Macherey group reported that using the BP strategy with symmetric pulse could not provide enough evidence for polarity behavior, whereas with applying asymmetric pulse, polarity effects were observed (Macherey and Carlyon, 2010; Macherey et al., 2011).

### Effect of Cochlear Implant Array

Commercially available CI arrays are generally produced in two types: lateral and perimodiolar arrays. In a lateral CI system, electrodes are located farther from the modiolus wall than in a perimodiolar array. To investigate the impact of electrode modiolus wall distance, we used both CI systems in a realistic model as reported in manufacturer data. Consistent with Schvartz-Leyzac et al. (2020), who observed that thresholds are directly proportional to electrode modiolus axis distance, our results indicate that lateral array needed larger threshold values compared with perimodiolar array.

However, owing to the fact that in a lateral array, the distance between channels is larger than in a perimodiolar array, we placed an additional electrode between two investigated existed electrodes. In addition, in perimodiolar system, we used the multipolar strategies with one gape in between channels (BP + 1, TP + 1, and PTP + 1) to have the channel distances as similar as possible in both CIs. Our result showed that reducing the channel distance in lateral CI affected only the threshold ranges and not the overall behavior of the thresholds. Consequently, we observed a significant increase in the threshold values due to the current shunt effect. Subsequently, increasing the channel distances in the perimodiolar CI led to a significant decrease in threshold values. Interestingly, in perimodiolar array, the threshold variation between cochlear turns slightly decreased due to wider electric field by setting one electrode gap between channels, which is in line with previous studies that reported variation in thresholds across the CI strongly depends on the expanse of the electric field (Bierer, 2007; Bierer et al., 2011; Bierer and Nye, 2014). Therefore, our results suggest that while the distance between electrodes alters the threshold ranges, the modiolus wall distance strongly affects threshold variations and consequently polarity ratios, not only across the array but also between degeneration levels when multipolar strategies are applied, as some clinical studies reported that the distance between electrode to modiolus wall impacts detection thresholds in multipolar strategies (Long et al., 2014; Mesnildrey et al., 2020).

On the other hand, similar to Long et al. (2014), who reported no significant difference in threshold ranges across the array in MP strategy, we also found analogous threshold ranges in both CIs, not only across the array but also between different degeneration levels when applying MP strategy. Consequently, we could not find any effect of electrode modiolus distance on polarity behavior in MP strategy, as also seen in the study by Jahn and Arenberg (2019a) that used MP strategy and reported that electrode modiolus wall distance does not affect polarity behavior.

### Perceptual Efficacy

Our study investigated the impact of pulse types, stimulation strategies, and electrode distance to modiolus on thresholds, spike initiation sites, and polarity sensitivity for normal and degenerated ANFs. However, the perceptual efficacy of the artificially generated spiking patterns depends essentially also on the population size of spiking ANFs per active channel. Frequency information transmitted by a small group of ANFs may be lost already during the first neural processing in the cochlear nucleus. Therefore, the focus on a stimulation strategy with sharp frequency selectivity is sometimes successfully replaced by sending the same (combined) input to two channels because of a poor ANF density in a specific frequency region of a patient or by using an implant with a low channel number (Saleh et al., 2013; Bierer and Litvak, 2016; Zhou, 2017). Electrically evoked compound APs recorded by modern CIs are of help to estimate the population size for each channel and intensity (Cullington, 2002; Abbas et al., 2004; Nehmé et al., 2014; DeVries et al., 2016). In addition, the amplitudes of the electrically evoked auditory brainstem response (Bierer and Faulkner, 2010; Bierer et al., 2011; Causon et al., 2019) reflect the frequency responses of each channel along the first processing centers. Both techniques can be applied to adapt our findings for individual patient data.

## Conclusion

Neural health investigation is of great concern for improving the functionality of the CIs which provides better perception for CI recipients. Polarity sensitivity is one technique for estimating neural health status; however, the clinical and computational outcomes based on polarity sensitivity have been contradictory over the past decades. We believe various parameters are crucial for studying polarity sensitivity associated with neural health. This study aims to investigate the effect of some of these parameters on polarity sensitivity. Our findings suggested the following:

(i) The asymmetric pulse shape is more suitable for studying polarity sensitivity when other parameters such as stimulation strategy, electrode distance to the modiolus wall, and cochlear turn are also considered.
(ii) MP as the default stimulation strategy in most CIs causes a broad electric field and uniform thresholds across the CI that negatively impact the polarity sensitivity and do not reveal helpful information on polarity behavior related to the ANF degeneration status.

(iii) In contrast, multipolar strategies demonstrated clear information associated with neural health when asymmetric pulse shape was considered. Therefore, a potential approach for estimating neural health is achieved by combining the asymmetric pulse shapes with multipolar strategies such as BP, TP, and PTP.
(iv) Finally, the distance between electrodes of the CI arrays and electrode distance to the modiolus wall affect threshold variations (consequently pulse polarity) only when multipolar strategies are applied, and the effect is not observed by MP strategy.

## Data Availability Statement

The original contributions presented in the study are included in the article/supplementary material, further inquiries can be directed to the corresponding author.

## Author Contributions

AH contributed to the conception, design of the study, design of FE model and computational model, data analysis, visualization, and manuscript writing and editing. SS contributed to the conception, design of FE model and computational model, data analysis, visualization, and manuscript writing and editing. AS-F contributed to administrate project, funding acquisition, and supervision. FR contributed to the conception, data analysis, article writing and revising, as well as supervision. All authors contributed to the article and approved the submitted version.

## Conflict of Interest

The authors declare that the research was conducted in the absence of any commercial or financial relationships that could be construed as a potential conflict of interest.

## Publisher’s Note

All claims expressed in this article are solely those of the authors and do not necessarily represent those of their affiliated organizations, or those of the publisher, the editors and the reviewers. Any product that may be evaluated in this article, or claim that may be made by its manufacturer, is not guaranteed or endorsed by the publisher.

## Footnotes

1
https://www.comsol.com

2
https://mathworks.com
